# Computational Modeling of Tensile Stress Effects on the Structure and Stability of Prototypical Covalent and Layered Materials

**DOI:** 10.3390/nano9101483

**Published:** 2019-10-18

**Authors:** Hocine Chorfi, Álvaro Lobato, Fahima Boudjada, Miguel A. Salvadó, Ruth Franco, Valentín G. Baonza, J. Manuel Recio

**Affiliations:** 1 MALTA-Consolider Team and Departamento de Química Física y Analítica, Universidad de Oviedo, E-33006 Oviedo, Spain; zahraoviedo@gmail.com (H.C.); a.lobato@ucm.es (Á.L.); mass@uniovi.es (M.A.S.); ruth@uniovi.es (R.F.); 2 Physics Department, University of Constantine 1, Constantine 25017, Algeria; boudjadafahima@yahoo.fr; 3 MALTA-Consolider Team and Departamento de Química Física, Universidad Complutense de Madrid, E-28040 Madrid, Spain; vgbaonza@ucm.es; 4 Institut Lumiere Matiere, Université Claude Bernard Lyon 1, CNRS, F-69622 Villeurbanne, France; 5 Instituto de Geociencias, IGEO, CSIC-UCM, E-28040 Madrid, Spain

**Keywords:** ideal strength, quantum-mechanical calculations, SiC, graphite, molybdenum disulfide, spinodal equation of state

## Abstract

Understanding the stability limit of crystalline materials under variable tensile stress conditions is of capital interest for technological applications. In this study, we present results from first-principles density functional theory calculations that quantitatively account for the response of selected covalent and layered materials to general stress conditions. In particular, we have evaluated the ideal strength along the main crystallographic directions of 3C and 2H polytypes of SiC, hexagonal ABA stacking of graphite and 2H-MoS${}_{2}$. Transverse superimposed stress on the tensile stress was taken into account in order to evaluate how the critical strength is affected by these multi-load conditions. In general, increasing transverse stress from negative to positive values leads to the expected decreasing of the critical strength. Few exceptions found in the compressive stress region correlate with the trends in the density of bonds along the directions with the unexpected behavior. In addition, we propose a modified spinodal equation of state able to accurately describe the calculated stress–strain curves. This analytical function is of general use and can also be applied to experimental data anticipating critical strengths and strain values, and for providing information on the energy stored in tensile stress processes.

## 1. Introduction

A clear understanding of the cohesive and mechanical properties of technological materials is of capital importance especially when applications are demanded in environments with hostile thermal, stress, and chemical conditions. Since the nature of the crystalline bonding networks is ultimately responsible for the response of the compounds to these external conditions, it is rewarding and necessary to investigate how macroscopic properties correlate with chemical interactions at an atomic level. Covalent and layered solids constitute two crystal families currently attracting interest in a variety of areas such as electronics and solar cell industries [1,2,3]. These compounds provide a good target to examine how changes in strong and weak interactions affect the observed elastic stability of materials. To this end, computer simulations constitute a practical research route to microscopically analyze strained structures of solids since geometries optimized by minimizing the crystal energy can be accurately obtained from first-principles electronic structure calculations under different stress conditions (see for example, [4]).

Within the above two families of compounds, silicon carbide (SiC), graphite and molybdenum disulfide (MoS${}_{2}$) are pertinent examples because, besides their genuine bonding networks, they are materials with a variety of applications in several technological sectors, such as new semiconductor devices, field effect transistors [1,2,5,6,7,8], lubricants [9,10], and components of solar cell panels [3]. In the manufacturing processes of these materials, mechanical failure may occur as a result of the stresses induced during the heating cycles to which the compounds are subjected. In addition, the simultaneous existence of covalent and van der Waals (vdW) interactions leads to preferential bi-dimensional and three-dimensional atomic arrangements in their crystalline structures that result in a high anisotropic response of these materials under variable stress conditions which is worth exploring.

The challenge consists in the accurate calculation of the limiting tension that these materials can support in particular directions. Considering perfect non-defective crystals, this maximum tension is known as the ideal or critical strength ($\sigma_{c}$) of the material for that direction. Both experimentally and theoretically, the evaluation of strain-stress curves constitutes the usual strategy to access this quantity, since after this critical point a catastrophic scenario emerges in the form of a crystal fracture or a phase transition. It then seems required to understand how the atomic level interactions correlate with the mechanism of failure in these environmental conditions and, if possible, anticipate the onset of the catastrophic scenario.

A number of theoretical studies using first-principles calculations, mainly employing density functional theory (DFT) [11,12], have permitted a quantitative evaluation of the critical strength of various materials (see [13,14,15] and references therein) showing that the effect of multi-axial stress obviously depends on the atomic species involved [16,17,18]. However, to the best of our knowledge, none of these studies have addressed the description of the observed or calculated stress–strain data by means of analytical functions as normally happens in high-pressure and related fields. Such equations of state would open the possibility of anticipating critical values for the strength and strain of materials without reaching the instability condition. In this regard, it is pertinent to recall the spinodal equation of state (SEOS) [19]. This analytical function was designed to describe the high-pressure behavior of condensed matter using as a reference state the onset of elastic instability. It has been successfully applied not only to the description of experimental and theoretical pressure-volume data, but also to the pressure evolution of one dimensional unit cell parameters [20]. Along with this fact, the SEOS is particularly well suited for the description of both experimental and theoretical stress–strain data derived from variable stress tensile conditions since, in the limit, these conditions precisely lead to the elastic instability of the material, i.e., the reference state for this analytical equation of state (EOS).

In this study, we performed DFT calculations to obtain the critical strength of 3C and 2H polytypes of SiC, graphite and 2H-MoS${}_{2}$ along their main crystallographic directions, with and without superimposed transverse stress conditions. The results are analyzed in terms of the density of chemical bonds and atomic interactions in the investigated directions of these materials. We are particularly interested in general analytical functions able to represent the behavior of different types of compounds under these tensile conditions and to reproduce the critical parameters. To this end, we propose a new SEOS form that uses the critical strain as the reference state, and that can be easily used to fit both the experimental and calculated stress–strain data.

Our paper is divided in three more sections. In the next section, we present the computational details of the electronic structure calculations and the algebra related with the new EOS. Section 3 contains the results and the discussion and is divided into three subsections, devoted, respectively, to the equilibrium properties of the four compounds, the stress–strain calculated curves, and the energetics and Young moduli derived from the proposed SEOS. The paper ends with a summary of our main findings.

## 2. Computational Details

### 2.1. Electronic Structure Calculations

First-principles electronic energy calculations and geometry optimizations under the Kohn–Sham DFT framework of 3C and 2H polytype structures of SiC, ABA stacking of graphite and hexagonal 2H-MoS${}_{2}$ are carried out with the ABINIT code [21,22] using the Perdew–Burke–Ernzerhof (PBE) exchange-correlation functional [23]. In order to take into account van der Waals forces, the correction (DFT-D2 ) to the exchange-correlation term, as proposed by Grimme [24], is used for graphite and MoS${}_{2}$. Although this pairwise approach does not capture many-body effects inherent to van der Waals interactions (see for example [25,26,27]), it has been proven to be accurate enough to determine optimized geometries involving the length scale (Å) of the tensile phenomena explored in this work. The so-called FHI atomic plane wave pseudopotentials [28] are adopted, while cut off energies and Monkhorst–Pack grids [29] are set to 1000 eV and 6 × 6 × 6 and 6 × 6 × 4 for 3C-SiC and 2H-SiC respectively, 1200 eV and 6 × 6 × 3 for graphite, and 400 eV and 6 × 6 × 2 for 2H-MoS${}_{2}$. Atomic positions were optimized until the total energy converged within 0.1 meV. At the same time, all the strain components (except in the applied loading direction) were optimized so that the corresponding stress components turned out to be within 100 MPa from a predetermined value. The Broyden–Fletcher–Goldfarb–Shanno minimization scheme (BFGS) [22] was used. In this way, tensile-strain curves under controlled normal stress were obtained. Ideal strength (critical strength from now on) was determined as the maximum value of tensile stress before the lattice loses stability and the forces diverge. Multi-axial stress calculations have been performed superimposing a transverse stress to the chosen stress direction. Atomic positions and movements through the different paths are analyzed using the visualization program for structural models (VESTA code) [30].

For the cubic 3C-SiC polytype, we calculate how the stress increases along the [100], [110] and [111] symmetry directions. For the hexagonal 2H-SiC polytype, and graphite and 2H-MoS${}_{2}$ layered crystals, calculations were performed along the inter-plane direction ([001]) perpendicular to the layers, and two in-plane directions, one containing nearest neighbors ([120], so-called zigzag direction) and the other connecting next nearest neighbors ([100], so-called armchair direction).

The stress tensor is calculated in ABINIT as the derivative of the total energy with respect to the strain tensor. The strain tensor, ϵ, can be calculated from the relation between the strain-free lattice vector of a given atom μ, $\vec{r_{\mu }}$, and its strained lattice vector, $\vec{r_{\mu }^{\prime }}$, as follows [31]:

(1) $$r_{\mu }^{\prime \alpha }=r_{\mu }^{\alpha }+\sum_{\beta =1}^{3}\epsilon_{\alpha \beta }r_{\mu }^{\beta },$$

where the α and β symbols denote the Cartesian components.

In the calculation of the second-order elastic constants in these cubic and hexagonal lattices, we follow an energy–strain scheme (see [32,33]). The lattice was first relaxed to achieve a zero stress state and then strains were applied by multiplying the lattice vectors by the strain matrix. For a lattice initially under no stress, and using Voigt notation, the energy of the strained lattice can be expressed around the equilibrium position as:

(2) $$E=E_{0}+\frac{V_{0}}{2}\sum_{i,j}C_{ij}\epsilon_{i}\epsilon_{j},$$

where $E_{0}$ and $V_{0}$ are, respectively, the energy and the volume of the unstrained lattice. There are three independent elastic constants for the cubic lattice ($C_{11}$, $C_{12}$, $C_{44}$) and five independent elastic constants ($C_{11}$, $C_{12}$, $C_{33}$, $C_{13}$, $C_{44}$) for the hexagonal one, thus three and five sets of finite strains were applied respectively. For each case, eleven equally-spaced strain values were applied between −0.05 and 0.05. The elastic constants were obtained from fitting a quadratic equation to the energy–strain calculated data points. The bulk modulus $B_{0}$ for each structure was calculated using its relationship with the elastic constants.

### 2.2. Spinodal-Like Stress–Strain Equation of State

From a thermodynamic point of view, the elastic stability limit of a solid at athermal conditions is defined by the point where the second derivative of the internal energy with respect to the volume becomes zero. At the corresponding pressure, also named as the spinodal pressure ($p_{\mathrm{sp}}$), the bulk modulus (*B*) of the substance tends to zero, and therefore any restoring force given by the chemical bonds is overcome, leading to a crystal rupture or a phase transition [34].

The spinodal locus has been considered as an excellent reference to describe the thermodynamic behavior of solids under high pressure conditions [19,35]. Polymers, metals, covalent and ionic crystals have been analyzed showing that their *p*-*V* data is accurately and universally represented through the spinodal constrain. This follows from the fact that along a given isotherm, the isothermal bulk modulus depends on the pressure through the following universal relation [36,37]:

(3) $$B=B^{★}{\left(p-p_{\mathrm{sp}}\right)}^{\beta },$$

where $B^{★}$ and β are, respectively, the amplitude and the pseudocritical exponent that characterize the pressure behavior of the isothermal bulk modulus.

The spinodal equation of state has not been used only in its volumetric form. For instance, Francisco et al. [20] studied the evolution under isotropic compression of the lattice parameters of rutile TiO${}_{2}$, showing that a one dimensional (1D) spinodal equation of state (1D-SEOS) can reproduce accurately their pressure dependence. To that, the authors define a linear bulk modulus, or equivalently a directional Young modulus ($Y_{I}$, I specifies the direction), and applied the universal relation of Equation (3). Considering both the physical significance and the directional behaviour of this spinodal-like equation of state, in this article we introduce a 1D-SEOS to analytically describe the stress–strain curves associated with tensile stress phenomena. Indeed, under directional stretching, the critical strength attained along the stress–strain curve corresponds to the spinodal stress limit, $\sigma_{\mathrm{sp}}$. The later parameter accounts for the maximum engineering stress at which the solid breaks, and therefore, represents the elastic limit of the material. Furthermore, at this spinodal point the directional Young modulus $Y_{I}$ has a value of zero, pointing out that there is no material resistance to a phase transition or rupture. Notice that these two parameters ($\sigma_{\mathrm{sp}}$ and $Y_{I}$) are also the one-dimensional analogs of the spinodal pressure and the bulk modulus. Consequently, from this perspective, the spinodal constrain is clearly fulfilled. Accordingly, the stress dependence of $Y_{I}$ can be accurately described with an amplitude factor $Y_{I}^{★}$ and a pseudocritical exponent γ following an equivalent power law form as Equation (3), and taking into account the engeneering convention of signs (σ is positive for tensile and negative for compressive stress):

(4) $$Y_{I}=Y_{I}^{★}{\left(\sigma_{\mathrm{sp}}-\sigma \right)}^{\gamma }.$$

Under these premises, an analytical stress–strain EOS can be derived. As the Young modulus is thermodynamically defined as the derivative of the stress with respect to the strain, the simple integration of Equation (4) leads to the following expression for a directional tensile curve:

(5) $$\sigma =\sigma_{\mathrm{sp}}-{\left\{Y_{I}^{★}\left(1-\gamma \right)\left(\epsilon_{\mathrm{sp}}-\epsilon \right)\right\}}^{1/(1-\gamma )}.$$

Equation (5) provides an analytical relationship between the stress and the strain along a particular direction of a crystalline solid involving four characteristic parameters. However, it must be emphasized that only three are independent since the spinodal strength, the spinodal strain and the amplitude factor are related realizing that no strain is present at σ = 0:

(6) $$Y_{I}^{★}\left(1-\gamma \right)=\frac{\sigma_{\mathrm{sp}}^{\left(1-\gamma \right)}}{\epsilon_{\mathrm{sp}}}.$$

Using this expression in Equation (5), we arrive at our final stress–strain 1D-SEOS:

(7) $$\sigma =\sigma_{\mathrm{sp}}\left(1-{\left(\frac{\epsilon_{\mathrm{sp}}-\epsilon }{\epsilon_{\mathrm{sp}}}\right)}^{\frac{1}{1-\gamma }}\right).$$

An interesting feature of the proposed stress–strain SEOS is that it can be also expressed analytically in its energy form. In fact, considering the isotherm at 0 K and neglecting zero point vibrational contributions, the stress is related to the internal energy *E* and the zero-pressure volume $V_{0}$ by means of [38]:

(8) $$\sigma =\frac{1}{V_{0}}\frac{dE}{d\epsilon }.$$

Consequently, the integrated energy–strain SEOS is:

(9) $$E_{\mathrm{sp}}-E=V_{0}\sigma_{\mathrm{sp}}\left(\epsilon_{\mathrm{sp}}-\epsilon \right)-V_{0}\frac{\left(1-\gamma \right)}{\left(2-\gamma \right)}{\frac{\sigma_{\mathrm{sp}}}{\epsilon_{\mathrm{sp}}}}^{\frac{1}{1-\gamma }}{\left(\epsilon_{\mathrm{sp}}-\epsilon \right)}^{\frac{2-\gamma }{1-\gamma }},$$

where $E_{\mathrm{sp}}$ is the internal energy of the solid at the spinodal strain, or equivalently the spinodal energy. This quantity must be understood as the energy needed to separate the crystallographic planes perpendicular to the stress–strain direction, and therefore to overcome the interatomic forces. Moreover, the spinodal energy can be expressed in terms of the spinodal stress and spinodal strain once we set to zero, the internal energy at zero strain:

(10) $$E_{\mathrm{sp}}=V_{0}\epsilon_{\mathrm{sp}}\left(\sigma_{\mathrm{sp}}-\frac{1-\gamma }{2-\gamma }\right).$$

An important feature of our current spinodal stress–strain EOS is that the spinodal energy gives us the opportunity to connect the mechanical parameters along a given tensile direction with the cohesive interatomic interactions.

Some words of caution on the notation should be given. First, $\sigma_{c}$ and $\sigma_{\mathrm{sp}}$ both represent the critical or ideal strength of the material along a given direction. The first symbol is obtained from ($\epsilon_{i}$,$\sigma_{i}$) calculated or experimental data, whereas the second one comes from our 1D-SEOS fittings as we discuss later. The same applies to $\epsilon_{c}$ and $\epsilon_{\mathrm{sp}}$. Second, in our static simulations (zero temperature and zero point energy contributions neglected), the internal energy of the system *E* is reduced to the electronic energy obtained in our DFT calculations. Finally, the symbol *E* is often used in other works to design the Young modulus. To avoid confussion, here we have chosen $Y_{I}$ for the directional Young modulus.

### 2.3. Spinodal Equation of State Fittings

The versatility of the proposed 1D-SEOS allows us to fit the Young modulus-stress (Equation (4)), stress–strain (Equation (7)), and energy–strain (Equation (10)) data. Since the spinodal hypothesis is based on the assumption that the universal relationship given in expression Equation (3) can accurately describe the stress dependence of the directional Young modulus, it becomes first necessary to examine if the proposed power law can fit the calculated data, in a reliable manner. To minimize numerical errors induced by the second strain derivative of the energy involved in the $Y_{I}-\sigma$ curves, a linear interpolation of the computed electronic energy has been performed. In all the cases, adjusted R-squares for the $Y_{I}$-σ curves lie in the range between 0.97 and 0.99 and residuals are equally distributed between negative and positive values with a percentage of deviation lower than 7%. In order to test the reliability of our proposed 1D-SEOS, the pseudocritical exponent and the critical strength and critical strain have been used as fitting parameters to analytically construct the stress–strain curves and energy–strain curves for all the directions and materials studied in this work according to the expressions derived in Section 2.2. Successfully, we obtain that the differences between the analytical curves and the calculated data are always below 1%. A summary of the fitting parameters are presented in Table 1.

As we can see in Table 1, the γ parameter lies inside the 0.41 ± 0.12 interval, depending on the crystal and the direction considered. These γ values are much lower than the universal β value of 0.85 assumed by Baonza et al. for the volumetric compression of solids [19]. Such a difference is attributed to the fact that we are in the stretching region in this case. Indeed, Brosh et al. [39] studied the dependence of the pseudocritical exponent as a function of the reduced volume both in the compressive and expansive regimes. These authors conclude that while the universal pseudocritical exponent of 0.85 accurately describes the solid under high and moderate pressure, the exponent goes down to the value of 0.5 in the case of the negative pressure regime, which is within the range of the results obtained in our spinodal stress–strain equation of state.

## 3. 3C-SiC, 2H-SiC, Graphite and 2H-MoS${}_{2}$: Results and Discussion

### 3.1. Bulk Properties

This subsection is restricted just to the summary of the equilibrium structural and elastic data of the four structures. Computed lattice constants, bulk moduli and elastic constants are collected in Table 2 along with experimental and other calculated values. Overall, our results are found to be in good agreement with the reported observed data, showing only slight differences due to the overestimation of the lattice constants and underestimation of the elastic constants inherent to the generalized gradient approximation (GGA) level of calculation. The introduction of the DFT-D2 correction, which is intended to take into account the vdW inter-layer interactions, leads our results for graphite and molybdenum disulfide to be in good agreement with the experiments and improves in general other previous local density approximation (LDA) or GGA results. In addition, the controversial $C_{12}$ parameter in 2H-MoS${}_{2}$, the higher discrepancy (less than 20%) is found in our calculation of $C_{11}$ in graphite (see Table 2). We attribute this deviation to the above tendency of GGA results. Regarding $C_{12}$ in 2H-MoS${}_{2}$, the situation is different. The discrepancy between the negative value reported in the experimental paper of Feldman [40] and the positive one obtained when the D2 Grimme correction is included in the calculations was discussed by Peelaers and Van de Walle [10]. We only notice here that $C_{12}$ was not directly measured but derived by Feldman using linear compressibilities reported in other works. Further details can be found in [10]. Overall, our calculated equilibrium properties provide the necessary reliable basis to undertake tensile stress simulations.

### 3.2. Ideal Strength with and without Transverse Stress

**Figure 1:**
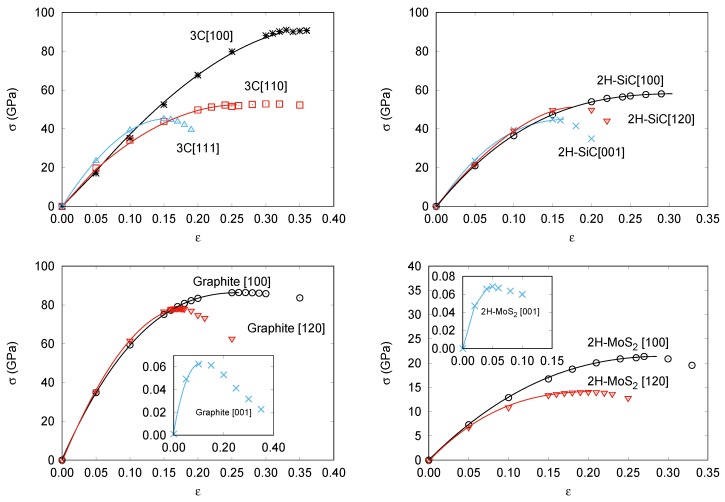
Calculated strain-stress curves without transverse stress for: 3C-SiC (**top left**), 2H-SiC (**top right**), Graphite (**bottom left**), and 2H-MoS${}_{2}$(**bottom right**).

This subsection is devoted to the calculation of the strain-stress curves of the four structures considered in this study. First, we collect in Figure 1 the results under vanishing transverse stress. For 3C-SiC and 2H-SiC, calculated points are very similar to those reported by Umeno, Kubo, and Nagao [42]. For graphite, our in-plane stress–strain curves show maxima at similar strain values to those reported by Liu et al. [48] for graphene, although we compute critical strengths along these directions around 25 GPa lower than in their work. This is due in part to differences between LDA (Liu et al.) and GGA (ours) levels of calculation, and on the other hand, to differences in the system, single sheet (graphene) and the bulk (graphite). To the best of our knowledge, the corresponding curve for the *c* direction has not been reported so far. Analogously, we have not found previous strain-stress curves along this direction for bulk 2H-MoS${}_{2}$, whereas for the in-plane directions the previous reported studies refer to single- or few-layer 2H-MoS${}_{2}$ [54,55]. These results indicate a noticeable decreasing of $\sigma_{c}$ as the size of the slab increases, which is also the expected trend according to our calculations.

It is usual to refer to the chemical bonding network to interpret, at an atomic level, the differences in the strain-stress curves between compounds and/or directions. Without being strictly quantitative, while keeping the basic chemical meaning, a simple and practical indicator able to account for the majority of these differences is proposed as follows. Each chemical bond in the unit cell is described by a vector connecting its two bound nearest-neighbor atoms. The projection of this vector along the corresponding tensile direction is evaluated and the sum calculated over all the bonds in the unit cell is defined as the total effective bond length (EBL) associated to that direction. The two main structural effects induced in the chemical bonds by the tensile strain (changes in bonding lengths and angles) are essentially captured in this parameter. EBL values exhibit the expected trend always increasing as the strain increases up to the stability limit.

Figure 1a shows that in 3C-SiC the slopes in the low strain region are nearly equal regardless of the direction. However, the maximum stress value strongly depends on the direction of the deformation with a critical strength nearly twice as large along the [100] axis ($\epsilon_{c}$ = 0.35 and $\sigma_{c}$ = 91 GPa), as that found for [110] ($\epsilon_{c}$ = 0.30 and $\sigma_{c}$ = 53 GPa) and [111] ($\epsilon_{c}$ = 0.15 and $\sigma_{c}$ = 45 GPa). We notice that along [100] all tensile forces are equally distributed over the Si-C bonds. This is in contrast to the tension along the [110] and [111] directions. For example, in the latter, one of the four C nearest neighbors of a given Si atom stand along the same [111] direction and the corresponding Si-C bond suffers a pure stretching, whereas the stretching of the other three Si-C bonds is not so effective and involves bond angle modifications upon the tensile strain along the [111] direction. At zero strain, the previously defined EBL parameter already has a value roughly twice greater for the [100] direction (17.5 Å) than for the [110] (9.3 Å) and [111] (9.5 Å) directions. Thus, although the order between the [100] and [111] directions is not captured considering just the equilibrium structure, the EBL parameter catches the essential difference between the [100] direction and these two other directions.

The stress–strain curves during uniaxial tension with vanishing transverse stress in 2H-SiC are shown in Figure 1b. Slopes in the low strain (harmonic) region are almost exactly equal whereas the maximum stress value strongly depends on the direction of the deformation. The stress–strain relation in 2H-SiC [001] ($\epsilon_{c}$ = 0.15 and $\sigma_{c}$ = 45 GPa) and 3C-SiC [111] are nearly identical. It is so because of the similarity of the lattice planes normal to the stress direction, and so are the curves of 2H-SiC [100] ($\epsilon_{c}$ = 0.29 and $\sigma_{c}$ = 58 GPa) and 3C-SiC [110]. The stress–strain relation in 2H-SiC along [120] shows intermediate values ($\epsilon_{c}$ = 0.20 and $\sigma_{c}$ = 50 GPa). Again, these values correlate with the effective Si-C bond lengths along the corresponding directions. Calculated EBL values in Å for the [100], [120] and [001] are, respectively, 21.3, 16.8, and 12.3, following the same trend as $\sigma_{c}$ and in agreement also with previous interpretations in terms of next-nearest Si-C interactions by Umeno et al. [42].

In Figure 1c,d, the responses of graphite and 2H-MoS${}_{2}$ to tensile stress along the [100], [120], and [001] directions are displayed. Here, the laminar nature of these two compounds is clearly revealed by the very low critical strength values along the *c* axis ($\epsilon_{c}$ = 0.13 and $\sigma_{c}$ = 0.063 GPa in graphite and $\epsilon_{c}$ = 0.05 and $\sigma_{c}$ = 0.069 GPa in 2H-MoS${}_{2}$) which is in concordance with the weak van der Waals nature of the inter-layer interaction. At low strains, the in-plane graphite strains reveal an isotropic 2D elastic behavior in good agreement with previous DFT calculations [56]. At large in-plane strains, the lattice layers start to behave anisotropically and the critical stress along the next-nearest-neighbor [100] direction ($\epsilon_{c}$ = 0.26 and $\sigma_{c}$ = 86 GPa in graphite and $\epsilon_{c}$ = 0.27 and $\sigma_{c}$ = 22 GPa in 2H-MoS${}_{2}$) becomes greater than that along the nearest-neighbor [120] direction ($\epsilon_{c}$ = 0.11 and $\sigma_{c}$ = 78 GPa in graphite and $\epsilon_{c}$ = 0.20 and $\sigma_{c}$ = 14 GPa in 2H-MoS${}_{2}$). Expected differences between stronger C–C than Mo–S intralayer bonds are also clearly manifested when comparing these data.

For all directions and structures, we now analyze new results coming from the proposed analytical 1D-SEOS. All the curves in the four panels of Figure 1 were obtained from the 1D-SEOS fittings to the calculated strain-stress data. The performance of the 1D-SEOS is apparent and allows us to derive with confidence critical stress and critical strain values from the corresponding fitting parameters $\sigma_{\mathrm{sp}}$ and $\epsilon_{\mathrm{sp}}$, respectively. We have checked that the trends and specific values of these two key parameters compare with high accuracy with our first-principles computed numerical values (see Table 1). Thus, we arrive to this interesting conclusion: the 1D-SEOS of Equation (7) is an appropriate analytical function for describing stress–strain data.

**Figure 2:**
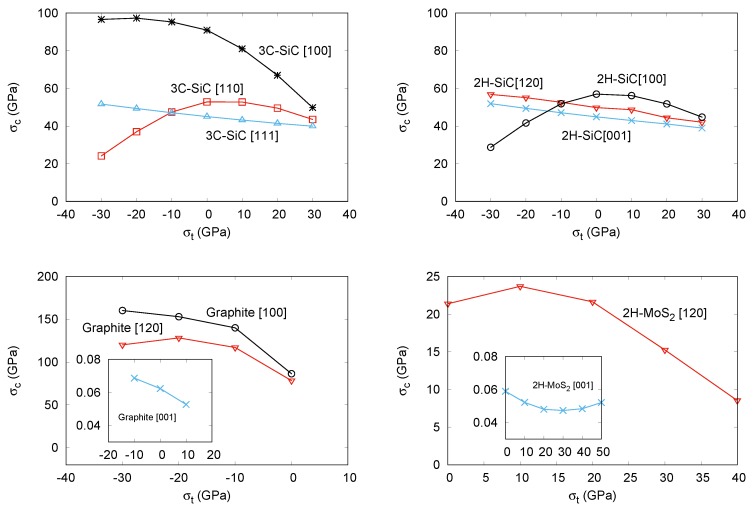
Calculated critical stress-transverse stress curves for: 3C-SiC (**top left**), 2H-SiC (**top right**), Graphite (**bottom left**), and 2H-MoS${}_{2}$(**bottom right**).

We noticed earlier that multi-load conditions may be present in manufacturing processes, combining thermal effects and epitaxial growth. As a particular situation of these conditions, in a second round of simulations, we have studied the effects of superimposing transverse stress (both compressive and tensile) on the previous tensile directions for the four structures. We detected convergence problems in some simulations that have hindered the calculations in the compressive (negative) transverse stress range in 2H-MoS${}_{2}$, and also along the [100] direction in the positive range of this compound. Based on previous resuls in other covalent systems [42], the expected trend is a decreasing of the critical strength as we increase the superimposed transverse stress from negative to positive values. In fact, this is the computed behavior for the majority of situations we have studied. For example, the critical strength $\sigma_{c}$ is lowered by the transverse stress $\sigma_{t}$ in all the directions in 3C-SiC (except [110]), 2H-SiC (except [100]), graphite, and 2H-MoS${}_{2}$. In this two laminar compounds, we obtain just one value at the most negative transverse stress breaking the decreasing trend along the [120] direction. All these results are displayed in Figure 2 and are in complete agreement with the computed data in 3C- and 2H-SiC reported by Umeno et al. [42]. In general, the unexpected positive slope in the critical strength-transverse stress curve appears at compressive transverse stress values. In the tensile regime, all the directions and structures show a modulated lowering of the critical strength as the transverse tension increases. This fact is compatible with the overall weakening of the compounds as multi-load conditions are enhanced, or, in Umeno et al. words as due to the higher strain energy stored in the material. However, we would like to notice that the opposite behavior was also found by Sestak et al. [15] and Cerný et al. [18]. The increasing of the critical strength under sumperimposed positive lateral tensile stress obtained in their calculations might be due to the different nature of the chemical bonding network. These authors deal with metallic materials where directional bonds are not identified, thus preventing the use for example of our EBL parameter that we introduce in what follows.

Interestingly enough, we observed an equivalent behavior when we analyzed the computed EBL parameters. In all but the cases where we have detected an exception, the calculated effective bond length parameter at the critical strain condition decreases monotonically as we superimpose the transverse stress on the corresponding tensile strain direction. Thus, we found that the decreasing of the critical strength value correlates with the decreasing in the EBL parameter. For example, along the [111] direction in 3C-SiC, EBL continuously decreases from 11.00 Å at $\sigma_{t}$ = −30 GPa to 10.78 Å at $\sigma_{t}$ = +30 GPa. The corresponding values at the same transverse stress conditions for the [100] direction are 24.71 Å and 21.18 Å. Similar trends are found for the EBL parameter along the [120] and [001] directions in 2H-SiC. On the contrary, in those cases where negative transverse stresses induce an unexpected behavior, this EBL parameter also shows as increasing as the transverse stress increases, up to the condition of vanishing transverse stress. Thus, along [110] in 3C-SiC and [100] in 2H-SiC, the values of EBL at $\sigma_{t}$ = −30 GPa are, respectively, 10.94 Å and 26.08 Å, increasing up to 11.49 Å and 26.24 Å at $\sigma_{t}$ = 0 GPa, and finally decreasing to 10.97 Å and 24.13 Å at $\sigma_{t}$ = +30 GPa. The reason why a reduction in the critical strength occurs as compressive transverse is superimposed has been explained by the appearance of a thermodynamic competitive phase as the rock-salt structure in 3C-SiC [42]. Here, we also see that this reduction in the $\sigma_{c}$ also correlates with the fact that the effective Si-C bond lengths along the [110] and [100] directions in 3C-SiC and 2H-SiC, respectively, show lower values at the critical conditions when the compressed transverse stress is increased, thus correlating with the trend followed by the critical strength.

#### Other Outcomes of the Stress–Strain SEOS: Energetics and Directional Young Moduli

**Figure 3:**
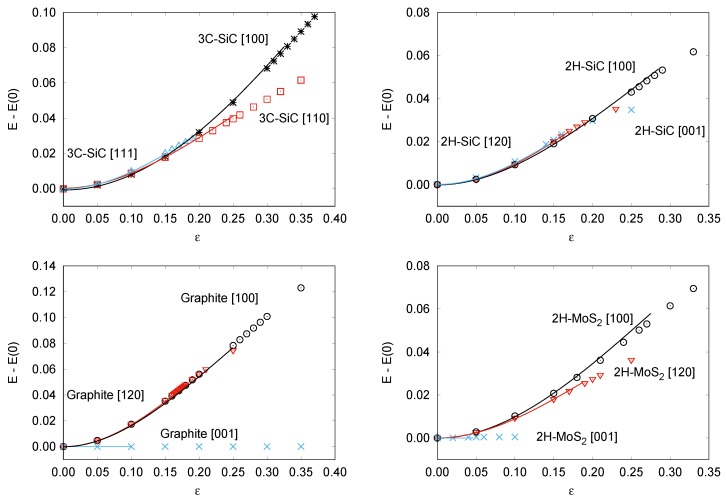
Calculated energy–strain curves for: 3C-SiC (**top left**), 2H-SiC (**top right**), Graphite (**bottom left**), and 2H-MoS${}_{2}$ (**bottom right**).

As stated in Section 2.3, our analytical scheme allows us to gather information, not only on the critical parameters, but also on the energetics of crystalline materials and on the Young moduli along specific tensile directions. From an experimental point of view, stress–strain data can be directly measured for particular directions whereas the corresponding energy–strain curves remain only accessible once an equation of state is proposed. Equation (10) displays how, by simple integration of our stress–strain 1D-SEOS, analytical energy–strain curves can be derived using data either from experiments or from computer simulations. In the previous subsection, we have shown that our calculated ($\epsilon_{i}$,$\sigma_{i}$) data points are well described by the proposed 1D-SEOS. Here, the integrated SEOS for all the directions and materials studied in this work are represented in Figure 3. The symbols correspond to the energy minima at selected strains obtained from our first-principles calculations. The calculated parameters associated with the integrated forms are collected in Table 3.

The analytical energy curves clearly reflect the good quality of the fittings (see Figure 3). Two parameters define the shape of each of these curves, $\epsilon_{\mathrm{sp}}$ and $E_{\mathrm{sp}}$. The first one, previously discussed in relation to the stress–strain curves (see Table 1), identifies the abscissa of the inflexion point, where the directional Young modulus vanishes. The ordinate of this point is $E_{\mathrm{sp}}$ (see Table 3) and correlates quite well with the critical/spinodal strength calculated along each of the directions explored for the materials under study in this work. The higher the strength, the higher the energy required to induce an elastic instability in the material. Not surprising, the highest values are obtained in 3C-SiC along the [100] direction and graphite along the [100] direction, just the same systems and directions where we had found the greatest values for $\sigma_{c}$ (and $\sigma_{\mathrm{sp}}$). $E_{\mathrm{sp}}$ values provide also information on the energy stored in the material due to the tensile stretching. For example, along the last two directions the energy stored is expected to be higher than along other directions with flatter energy–strain curves, as [001] directions in graphite and 2H-MoS${}_{2}$ (see Figure 3). Notice that for these two situations with the weakest cohesive interactions, values are so low (within the accuracy of the calculations) that only a limit value is given. Overall, we believe that these results evidence the utility of the energy–strain SEOS.

As regards the directional Young modulus, we can easily derive a simple expression at zero stress $Y_{I}$(0) involving the three parameters of the stress–strain 1D-SEOS by evaluating Equation (4) at zero stress:

(11) $$Y_{I}\left(0\right)=\frac{\sigma_{\mathrm{sp}}}{\epsilon_{\mathrm{sp}}\left(1-\gamma \right)}.$$

This parameter is discussed below.

In 3C-SiC, the directional Young moduli at zero stress are (in GPa) 396, 406, 478 GPa for the [100] [110] and [111] directions, respectively. These results are in concordance with the directional Young moduli calculated through the theory of representation surfaces [57]. For instance, in the case of the [111] direction

(12) $$Y_{111}={\left(S_{11}-\frac{2}{3}\left(S_{11}-S_{12}-\frac{1}{2}S_{44}\right)\right)}^{-1},$$

where $S_{11},S_{12}$, and $S_{44}$ are the compliance constants related to the elastic constants by:

(13) $$S_{11}=\frac{C_{11}+C_{12}}{\left(C_{11}-C_{12}\right)\left(C_{11}+2C_{12}\right)},S_{12}=\frac{-C_{12}}{\left(C_{11}-C_{12}\right)(C_{11}}+2C_{12}),S_{44}=\frac{1}{C_{44}}.$$

According to the data from Table 2, and using the above equations, the calculated value for $Y_{111}$(0) is 489 GPa in good agreement with the parameter obtained from our 1D-SEOS.

In this case, the elastic behavior of the cubic SiC polytype is not enterely isotropic and $Y_{I}$(0) slightly increases along the sequence [100] [110] and [111]. $Y_{I}$(0) provides a quantitative measure of the initial slope of the stress–strain curve, thus representing the resistance of the material to a tensile distortion along a particular direction at equilibrium. Under this perspective, the values of $Y_{I}$(0) in the [100], [110] and [111] series of 3C-SiC inform that the direction [111] offers the highest resistance to a strain stretching at zero stress. In 2H-SiC, the values of $Y_{I}$(0) point out that all the directions studied present similar resistance to distortion. Here, the solid behaves less anisotropically than in the case of the cubic polytype, expanding a narrower range of values, although both polytypes display similar zero stress Young moduli.

Let us finally conclude by analyzing these zero stress directional Young moduli in graphite and 2H-MoS${}_{2}$. Layered materials constitute a severe test for our model since weak and covalent interactions are simultaneously present. In both compounds, the van der Waals nature of the inter-layer interactions is revealed through the values of the directional Young modulus provided by the spinodal parameters. $Y_{001}$(0) values (in GPa) are as low as 0.99 and 2.40 for graphite and 2H-MoS${}_{2}$, respectively, in contrast with the values along the [100] and [120] directions which are, respectively, 748 and 728 for graphite, and 150 and 140 for 2H-MoS${}_{2}$. The latter values can be compared with the intra-layer Young modulus reported for graphite and MoS${}_{2}$ by other authors. For instance, for graphite goes from 700 to 1100 GPa ([56] and references therein), whereas for 2H-MoS${}_{2}$ the values range between 130 and 220 GPa [58,59,60] showing a good agreement with the results obtained in this work. At this point, it must also be emphasized that our Young modulus values reflect the expected different intralayer bond strengths between the C–C and Mo–S bonds, as we previously detected in the analysis of the 1D-SEOS parameters (see Section 3.2).

## 4. Conclusions

The critical strength of 3C- and 2H-SiC, graphite, and 2H-MoS${}_{2}$ were evaluated by means of first principles quantum-mechanical methodologies based on the DFT approximation. Both vanishing and superimposed transverse stress over uniaxial tensile strains were considered in order to evaluate the critical (ideal) strength of the four crystalline structures. The critical strength is found to depend on the particular crystallographic direction revealing the expected stronger mechanical anisotropy in the layered compounds. In graphite and molybdenum disulfide layers, after an isotropic behavior at the low strain regime, we observe a different behavior along the two in-plane directions, the critical tensile strength being smaller in the nearest-neighbor than in the next-nearest-neighbor direction. In these crystals, the lowest value of $\sigma_{c}$ is obtained in the *c*-direction as expected given the weak inter-layer vdW interactions. The critical tensile strength is generally decreased by the transverse tension. Reduction in the critical strength by large transverse compression occurs in some structures and orientations in concordance with an increase in the effective bond lengths in those conditions.

We present a new 1D-SEOS analytical function that was successfully applied to the computed strain-stress data points, and which can be also used to describe results from tensile stress experiments. The spinodal strain $\epsilon_{\mathrm{sp}}$ along with the corresponding spinodal stress $\sigma_{\mathrm{sp}}$ fitting parameters have been calculated for the two covalent and the two layered compounds. These parameters are identified with the critical strength and strain values provided they appear at the instability elastic limit. In addition, the integrated energy–strain SEOS reveals an interesting equation, enclosing information on the energy stored in the material along tensile processes and providing data on the required energy to reach the instability elastic limit.

## Figures and Tables

**Table 1:** One dimensional (1D) spinodal equation of state (1D-SEOS) parameters from the fittings to our computed stress–strain data. Units of $\sigma_{\mathrm{sp}}$ are GPa.

Material	Direction	γ	$\epsilon_{\mathrm{sp}}$	$\sigma_{\mathrm{sp}}$
3C-SiC	[100]	0.29	0.35	90.5
	[110]	0.49	0.30	52.3
	[111]	0.36	0.15	45.1
2H-SiC	[001]	0.36	0.15	44.9
	[100]	0.46	0.29	58.0
	[120]	0.34	0.17	50.7
Graphite	[001]	0.35	0.99	0.06
	[100]	0.53	0.26	85.8
	[120]	0.37	0.11	78.3
2H-MoS${}_{2}$	[001]	0.39	0.05	0.07
	[100]	0.38	0.27	21.4
	[120]	0.46	0.20	14.2

**Table 2:** Zero pressure lattice and elastic constants of 3C- and 2H-SiC polytypes, graphite and 2H-MoS${}_{2}$. All $B_{0}$ values calculated using Voigt elastic constants relationship.

		This Work	Calculated	Experimental
3C-SiC	*a*(Å)	4.39	4.34 [41], 4.38 [42]	4.34 [43]
	$C_{11}$(GPa)	341	390 [41], 385 [42]	352 [44]
	$C_{12}$(GPa)	130	134 [41], 128 [42]	140 [44]
	$C_{44}$(GPa)	224	253 [41], 264 [42]	233 [44]
	$B_{0}$(GPa)	200	219, 213	211
2H-SiC	*a*(Å)	3.085	3.05 [45], 3.09 [42]	3.076 [46]
	*c*(Å)	5.060	5.00 [45], 5.07 [42]	5.224 [46]
	$C_{11}$(GPa)	528	541 [45], 536 [42]	501 ± 4 [47]
	$C_{12}$(GPa)	112	117 [45], 78 [42]	111 ± 5 [47]
	$C_{33}$(GPa)	565	586 [45], 573 [42]	553 ± 4 [47]
	$C_{13}$(GPa)	52	61 [45], 31 [42]	52 ± 9 [47]
	$C_{44}$(GPa)	156	162 [45], 164 [42]	163 ± 4 [47]
	$B_{0}$(GPa)	228	238, 214	220
Graphite	*a*(Å)	2.521	2.451 [48]	2.464 [49]
	*c*(Å)	7.067	6.582 [50]	6.712 [49]
	$C_{11}$(GPa)	892	1118 [51]	1109 ± 16 [49]
	$C_{12}$ (GPa)	163	235 [51]	139 ± 36 [49]
	$C_{33}$ (GPa)	31	29 [51]	38.7 ± 7 [49]
	$C_{13}$ (GPa)	5	8.5 [51]	0 ± 3 [49]
	$C_{44}$ (GPa)	6	−2.8 [51]	5 ± 3 [49]
	$B_{0}$ (GPa)	240	307	281
2H-MoS${}_{2}$	*a*(Å)	3.19	3.16 [52]	3.163 [53]
	*c*(Å)	12.56	12.296 [52]	12.341 [53]
	$C_{11}$ (GPa)	220	218 [52]	238 [40]
	$C_{12}$ (GPa)	45	38 [52]	−54 [40]
	$C_{33}$ (GPa)	40	35 [52]	52 [40]
	$C_{13}$ (GPa)	16	17 [52]	23 [40]
	$C_{44}$ (GPa)	26	15 [52]	19 [40]
	$B_{0}$ (GPa)	75	68	57

**Table 3:** Energy and Young modulus parameters from the integrated stress–strain SEOS fittings.

Material	Direction	$Y_{I}$(0) (GPa)	$E_{\mathrm{sp}}$ (kJ/mol)
3C-SiC	[100]	396	219
	[110]	407	110
	[111]	478	50
2H-SiC	[001]	481	50
	[100]	437	142
	[120]	450	66
Graphite	[001]	0.99	<1
	[100]	746	201
	[120]	746	113
2H-MoS${}_{2}$	[001]	2.41	<1
	[100]	150	69
	[120]	140	153
